# Evaluation of the measurement accuracy and uncertainty of a solid‐state detector under diagnostic x‐ray beam conditions

**DOI:** 10.1002/acm2.14476

**Published:** 2024-07-19

**Authors:** Sho Maruyama, Hiroki Saitou, Toru Negishi, Michiharu Sekimoto

**Affiliations:** ^1^ Department of Radiological Technology Gunma Prefectural College of Health Sciences Gunma Japan; ^2^ Department of Medical Radiology Faculty of Medical Technology Teikyo University Tokyo Japan; ^3^ Department of Radiological Sciences Graduate School of Human Health Sciences Tokyo Metropolitan University Tokyo Japan; ^4^ Faculty of Medical Technology Niigata University of Health and Welfare Niigata Japan

**Keywords:** air kerma, diagnostic x‐ray, half‐value layer (HVL), measurement accuracy, solid‐state detector (SSD), tube voltage, uncertainty

## Abstract

**Objective:**

An accurate measurement of x‐ray beams is expected to reduce the uncertainties associated with estimating radiation risk to patients in clinical settings. To perform assessment tasks based on the readings of a solid‐state detector (SSD) using semiconductor technology, the characteristics of the detector should be elucidated. In this study, we evaluated the measurement accuracy of a new SSD under diagnostic x‐ray beam conditions in terms of air kerma, tube voltage, and half‐value layer (HVL). The performance of the SSD was then compared with those of reference instruments.

**Methods:**

The tube voltage was varied within the range of 50–120 kV in steps of 10 kV and the thickness and materials of additional filters were concurrently changed (several combinations were tested). In addition, the dose rate and energy dependence of the SSD were also investigated. These effects were analyzed based on statistical significance tests. Furthermore, the expanded uncertainties in the series of measurements were meticulously calculated.

**Results:**

The results showed average relative differences of −3.26 ± 1.33%, 0.44 ± 1.01%, and −2.60 ± 3.31% for air kerma, tube voltage, and HVL, respectively. Furthermore, air kerma did not exhibit any dependence on dose rate and energy, in contrast to tube voltage and HVL measurements.

**Conclusion:**

The measurement values of the SSD fall within the acceptable range of uncertainty, highlighting its measurement accuracy and reliability. Furthermore, based on the characteristics elucidated by this study, valuable insights are provided concerning the assurance of appropriate measurement values in clinical settings.

## 1 INTRODUCTION

Diagnostic x‐ray imaging plays an essential role in medical care. Although there are no established dose limits for medical exposure through justified examinations, the concept of a diagnostic reference level was introduced to optimize imaging doses.^1^, ^2^ Japan has established stringent regulations regarding the output management of x‐ray equipment used for medical purposes. These regulations mandate the management and recording of patients’ exposure levels.^3^ This emphasizes the importance of quality control (QC) of x‐ray equipment and the responsibility of the user for proper radiological diagnosis.^4^

The QC of x‐ray equipment has traditionally been performed using an ionization chamber (IC) and a direct‐connected tube voltage meter.^5^, ^6^ However, handling these instruments is not straightforward, and a certain level of experience is required to enhance accuracy. In addition, to ensure electrical safety, the guidelines recommend the use of calibrated and non‐invasive instruments instead of a tube voltage meter.^7^ Such instruments are transitioning to solid‐state detectors (SSDs), and highly accurate and multifunctional non‐invasive instruments using multi‐detector approaches are currently under development.^8^, ^9^, ^10^ These instruments are commonly used in clinical practice and have been noted for their utility, as they can measure multiple parameters in a single exposure.^11^, ^12^, ^13^, ^14^, ^15^

Recently, a new SSD with a reduced size and multisensor features was introduced, which can operate under a broad range of x‐ray irradiation conditions.^16^ Lin et al. have verified its accuracy in terms of half‐value layer (HVL). They reported that the error in the HVL measurements was within 0.5 mm Al compared to those obtained using an IC system.^17^ However, considering the application of these measurements to assess the radiation risk for patients in clinical settings, these data are not sufficient. The practicality of a new SSD is determined by understanding the accuracy and reliability of its readings through meticulous analysis. Therefore, we evaluated the performance of an SSD under diagnostic x‐ray beam conditions and comprehensively compared it with the results obtained using an IC and tube voltage meter.

## 2 METHODS

### 2.1 A mutual comparison between the SSD and the reference device

We verified the measurement accuracy of an ACCU‐GOLD+ (Radcal, California, USA) SSD^16^ and compared it with those of two reference instruments: 6‐cm^3^ stereotactic field diode IC‐type TW 34069, equipped with the dosimeter UNIDOS E (PTW, Freiburg, Germany), and the tube voltage meter AB‐2015E (TORECK, Kanagawa, Japan). For generating x‐rays, we employed the diagnostic x‐ray system DHF‐155H with a total filtration of 2.8 mm Al (Hitachi, Tokyo, Japan). The coefficient of variation for the exposure parameters in ten repeated measurements was below 0.005.^18^

**FIGURE 1:**
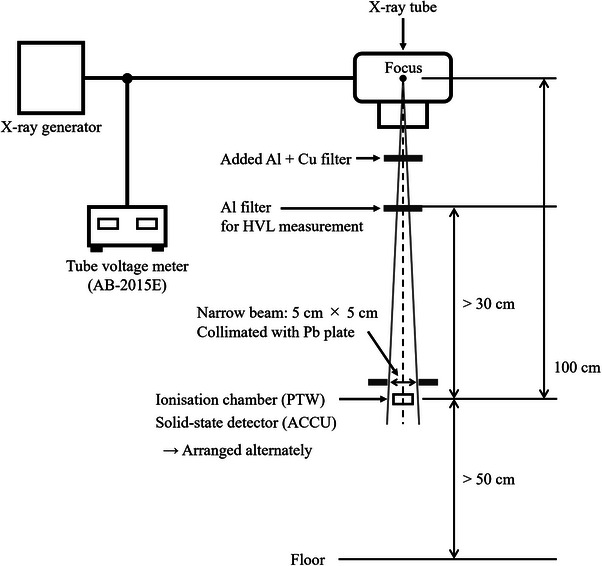
Overview of measurement arrangements.

The performances were compared in terms of air kerma, tube voltage, and HVL. Measurement instruments were arranged as shown in Figure 1, and all measurements were conducted under the following conditions: tube current of 200 mA, exposure time of 50 ms, and focus‐to‐detector distance of 100 cm. The irradiation field on the detector surface was 5 × 5 cm^2^, and the narrow beam was collimated by placing a 2‐mm‐thick Pb plate above the detector. The instruments were arranged considering the influence of backscattering and scattered radiation from the Al filter.^19^, ^20^

The tube voltage was varied within a range of 50−120 kV in steps of 10 kV. The thickness of the additional filters was changed concurrently to 0, 2, and 4 mm of Al and 2, 4, 6, and 9 mm of Al + 0.1 mm of Cu, and 56 patterns of conditions were examined. The values and their errors for each measurement were examined by varying the set tube voltage with the filter thickness as a parameter.

The air kerma obtained from the IC was determined by multiplying the measured value by the calibration constant and the temperature‐pressure correction factor; the air kerma for the ACCU was directly adopted from the obtained measurement value. The reference HVL of the IC was determined by following the commonly adopted method,^13^, ^21^ whereas the HVL of the SSD was assessed on basis of readings. Furthermore, the tube voltage values were assessed using the readings of each instrument (ACCU and voltage meter).

The relative differences (RDs) were calculated using reference instruments as standards. We then conducted statistical significance tests to investigate whether the RDs were influenced by the additional filter thickness. If normality (Shapiro–Wilk test) and homoscedasticity (Bartlett test) were confirmed in the data, the one‐way analysis of variance (ANOVA) test was used. If normality was not observed, the Kruskal–Wallis test was performed. If a significant RD was observed, post‐hoc Tukey's or Steel–Dwass tests were conducted, depending on the presence or absence of normality.

### 2.2 Verification of measurement uncertainty

To clarify the uncertainties in the series of measurements, we calculated the expanded uncertainties of the measurements using the reference instruments and ACCU for each evaluation item.^22^ The expanded uncertainty was determined by calculating all standard uncertainties and the coverage factor.

### 2.3 Dose rate and energy dependence of the SSD

To investigate the dose rate and energy dependence of the ACCU, we determined the correlation coefficients for the air kerma rate (dose rate) and HVL for the RDs for each measurement item and performed a non‐correlation test. The values used in these assessments were obtained under varying conditions, with the insertion of different filters.

## 3 RESULTS

### 3.1 Mutual comparison between the SSD and the reference instruments

#### 3.1.1 Measurements of air kerma

**FIGURE 2:**
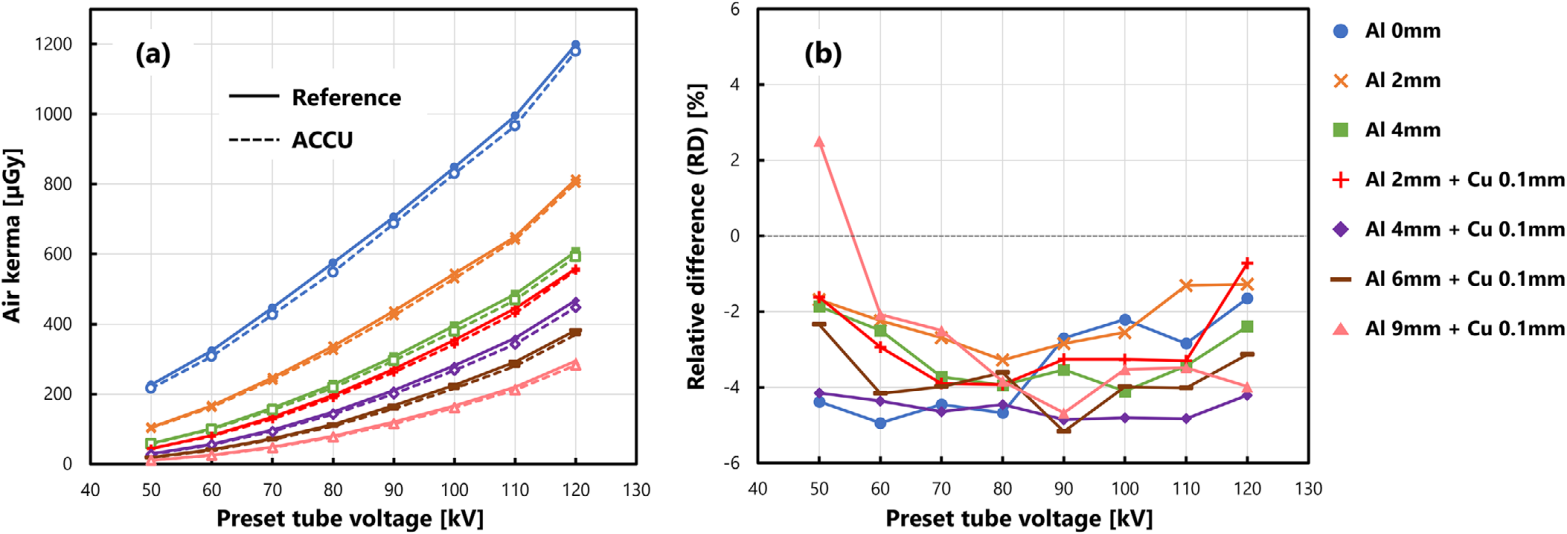
Results of air kerma measurement accuracy. (a) Comparison between the reference IC and the ACCU. The plot is color‐coded based on the filter type, as shown in (b). The solid and dashed lines represent the measurements from the IC and ACCU, respectively. (b) Relative differences in the ACCU with respect to the IC.

Figure 2a,b shows the air kerma measured at various preset tube voltages and the RDs, respectively. Compared to the IC, the ACCU tended to underestimate the air kerma, with an average value of −3.26 ± 1.33% and a quartile range of −4.15% to −2.50%.

For the RDs in air kerma, the Kruskal–Wallis test was conducted and the results indicated a significant difference due to the presence of additional filters ($p=7.93\times 10^{-4}<0.05$). Subsequently, multiple comparisons using the Steel–Dwass test revealed significant differences between Al 2 and Al 4 + Cu 0.1, Al 4 and Al 4 + Cu 0.1, and Al 2 + Cu 0.1 and Al 4 + Cu 0.1 filters.

#### 3.1.2 Measurements of tube voltage

**FIGURE 3:**
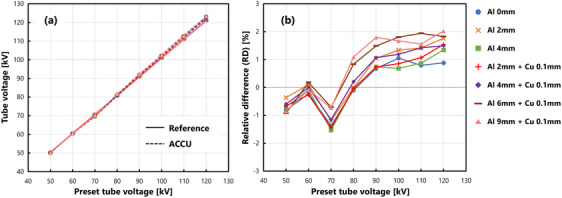
Results of tube voltage measurement accuracy. (a) Comparison between the reference voltage meter and the ACCU. The color code and line representation are the same as those in Figure 2. (b) Relative differences in the ACCU measurements with respect to the voltage‐meter measurements.

Figure 3a illustrates the measured results of the tube voltage for various preset tube voltages, and Figure 3b shows the RDs. As the preset tube voltage increased, the RDs tended to increase to positive values. However, the average value was 0.44 ± 1.01%, with a quartile range of −0.29% to 1.34%.

The results of Kruskal–Wallis test showed that there was no significant difference due to the additional filters (p=0.49>0.05).

#### 3.1.3 Measurements of HVL

**FIGURE 4:**
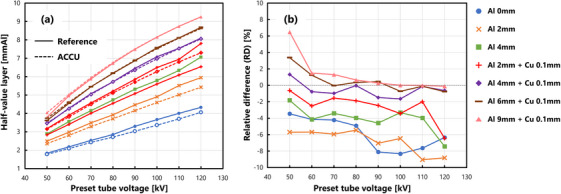
HVL measurement accuracy. (a) Comparison between the reference IC and the ACCU. The color code and line representation are the same as those in Figure 2. (b) Relative differences in the ACCU measurements with respect to the IC measurements.

Figure 4a shows the HVL measured under various preset tube voltages, and Figure 4b shows the RDs. An average of −2.60 ± 3.31% was observed with a quartile range of −5.05% to −0.09%.

The data for RDs in HVL were confirmed to be normal and homogeneous. Therefore, one‐way ANOVA was performed, which revealed a significant difference due to the presence of additional filters ($p=1.55\times 10^{-14}<0.05$). The results of Tukey's post‐hoc test for multiple comparisons indicated no significant differences for combinations of Al 0 versus Al 2 and Al 4, Al 2+Cu 0.1 versus Al 4, Al 4+Cu 0.1 versus Al 6+Cu 0.1 and Al 9+Cu 0.1, and Al 6+Cu 0.1 versus Al 9+Cu 0.1. By contrast, significant differences were observed for other combinations.

### 3.2 Verification of measurement uncertainty

Tables 1, 2, 3 present the budget sheets estimating the standard uncertainties of the reference instruments for each evaluation item. The factors contributing to the uncertainty varied depending on the measured items. The values include a mixture of percentages and those obtained from the minimum scale of the measuring devices. Hence, the differences are presented by specifying the units for clarity. The verification results indicated that there were no significant differences in uncertainties between the reference instruments and ACCU. This uncertainty was attributed primarily to the calibration of the measuring instrument.

**TABLE 1:** Budget sheet for the uncertainty estimation in air kerma measurements.

Uncertainty factor	Value		Type	Distribution	Divisor	Relative standard uncertainty
Calibration (chamber)	5	[%]	B	Normal	2	2.50
Resolution (chamber)	0.005	[µGy]	B	Rectangular	1.73	0.00
Geometrical arrangement	0.5	[mm]	B	Rectangular	1.73	0.06
Measurement results	0.31	[%]	A	Normal	1	0.31
Temperature	0.05	[°C]	B	Rectangular	1.73	0.01
Atmospheric pressure	0.05	[hPa]	B	Rectangular	1.73	0.03
Stability of the x‐ray system	0.07	[%]	A	Normal	1	0.07
Combined uncertainty [%]						2.52
Expanded uncertainty (*k* = 2) [%]						5.05
Round up value						**6.00**
ACCU*						
Expanded uncertainty (*k* = 2) [%]						**5.00**

*Note*: The PTW IC dosimeter was used as the measurement instrument. The uncertainty of ACCU measurements was transcribed from the technical data,^16^ which is marked with *.

**TABLE 2:** Budget sheet for the uncertainty estimation in tube voltage measurements.

Uncertainty factor	Value		Type	Distribution	Divisor	Relative standard uncertainty
Calibration (voltage meter)	1.5	[%]	B	Normal	2	0.75
Resolution (voltage meter)	0.05	[µGy]	B	Rectangular	1.73	0.04
Measurement results	0.113	[%]	A	Normal	1	0.31
Stability of the x‐ray system	0.07	[%]	A	Normal	1	0.07
Combined uncertainty [%]						0.81
Expanded uncertainty (*k* = 2) [%]						1.63
Round up value						**2.00**
ACCU**						
Expanded uncertainty (*k* = 2) [%]						**2.00**

*Note*: The tube voltage meter AB‐2015E was used as the measurement instrument. The uncertainty of ACCU measurements was transcribed from the technical data,^16^ which is marked with **.

**TABLE 3:** Budget sheet for the uncertainty estimation in HVL measurements.

Uncertainty factor	Value		Type	Distribution	Divisor	Relative standard uncertainty
Calibration (chamber)	5	[%]	B	Normal	2	2.50
Resolution (chamber)	0.005	[µGy]	B	Rectangular	1.73	0.00
Geometrical arrangement	0.5	[mm]	B	Rectangular	1.73	0.06
Measurement results	0.3	[%]	A	Normal	1	0.30
Temperature	0.05	[°C]	B	Rectangular	1.73	0.01
Atmospheric pressure	0.05	[hPa]	B	Rectangular	1.73	0.00
Stability of the x‐ray system	0.07	[%]	A	Normal	1	0.06
Al filter thickness	1	[%]	B	Normal	2	0.50
HVL linear interpolation	2	[%]	B	Normal	2	1.00
Combined uncertainty [%]						2.76
Expanded uncertainty (*k* = 2) [%]						5.51
Round up value						**6.00**
ACCU***						
Expanded uncertainty (*k* = 2) [%]						**5.00**

*Note*: The PTW IC dosimeter was used as the measurement instrument. The uncertainty of ACCU measurement was transcribed from the technical data,^16^ which is marked with ***.

### 3.3 Dose rate and energy dependence of the SSD

**FIGURE 5:**
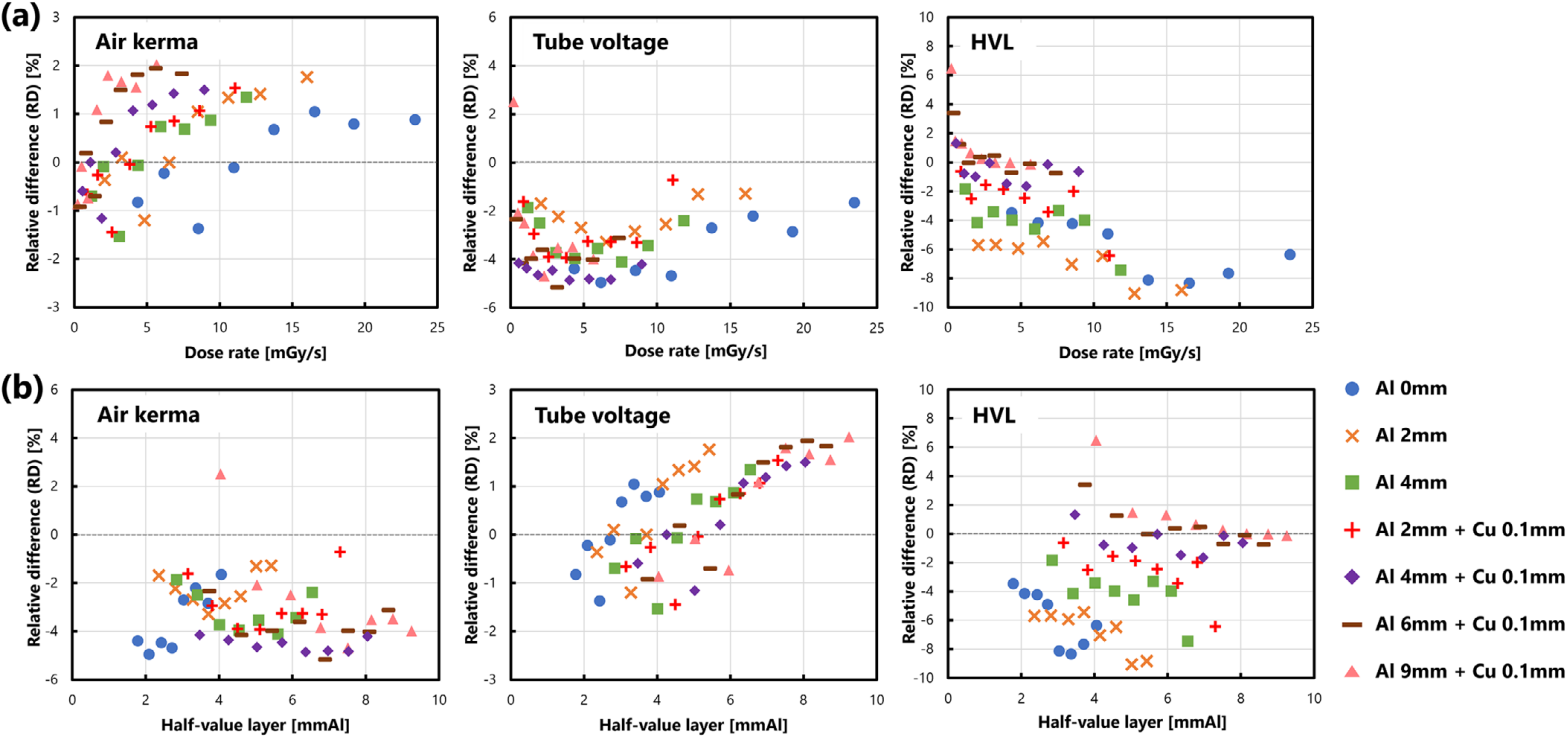
(a) Dose‐rate dependencies and (b) x‐ray energy dependencies in the ACCU measurements. Relative differences in air kerma, tube voltage, and HVL are shown respectively. The horizontal axis represents the ACCU measurements. The legend, shared across all subfigures, is shown on right side.

Figure 5a shows the RDs in each measurement item obtained using the ACCU for various dose rates. There was no correlation for air kerma, as evident from its coefficient of 0.18 (p=0.182>0.05). Further, we observed a positive correlation of 0.43 for tube voltage ($p=1.1\times 10^{-3}<0.05$), and a strong negative correlation of −0.74 for HVL ($p=9.0\times 10^{-11}<0.05$). Figure 5b illustrates the RDs for each measurement item when the HVL was varied as an index of energy dependence. Air kerma showed no correlation, as evident from the correlation coefficient of −0.22 (p=0.097>0.05). Tube voltage exhibited a strong positive correlation of 0.73 ($p=1.6\times 10^{-10}<0.05$), and HVL indicated a weak positive correlation of 0.35 (p=0.008<0.05). These results were obtained using the values shown in Figures 2, 3, 4.

## 4 DISCUSSION

### 4.1 SSD accuracy

#### 4.1.1 Air kerma measurements

The standard test conditions for semiconductor dosimeters are specified in IEC 61674.^23^ The reference radiation quality RQR5 mandates an HVL of 2.58 mm Al at a tube voltage of 70 kV.^24^ As illustrated in Figure 2, an approximately −4% RD was observed under a tube voltage of 70 kV, and a similar −4% error was observed in the voltage range of 50−80 kV. However, the RD tended to decrease when the preset tube voltage exceeded 90 kV. This can be attributed to the intrinsic sensitivity of semiconductors. The narrow range of RDs in air‐kerma measurements (−2% to −5%), biased toward the negative value side, suggests that the actual RD would be approximately 3% if the negative distribution was increased to 0% by accounting for the decrease in sensitivity (assuming that it can be calibrated using a correction factor). The expanded uncertainties for this measurement system (Table 1) implied that the RD was within the measurement uncertainty range, and the measurement accuracy of the ACCU was considered comparable to that of the IC.

#### 4.1.2 Tube voltage measurements

The tube‐voltage measurements from ACCU exhibited an RD that converged between 60 and 70 kV (Figure 3). As the point at 60 kV exhibited a narrower convergence width, it may appear similar to the calibration point for the ACCU. However, as semiconductor detectors are calibrated at 70 kV as per the RQR5 standards, it is reasonable to consider the convergence point at 70 kV as the reference. Although a slight energy dependence was observed in the higher voltage region, the expanded uncertainty in the tube voltages measured using the tube‐voltage meter was 2% for the reference^25^ and ACCU, as presented in Table 2. Therefore, the error introduced owing to this dependence was within the acceptable range of uncertainty. Hence, we concluded that the RD in tube‐voltage was not affected by the RD in air kerma because the energy was determined by multiple sensors in the detector.

#### 4.1.3 HVL measurements

Using the IC, the HVL was obtained based on the linear interpolation between two measurement points. Hence, an overestimation of approximately 1% was reported.^26^ Based on this result, the net RD was estimated as the value obtained by subtracting 1% from the RD. The trend of the observed RD was consistent with the results reported by Lin et al., who indicated that the difference between the HVL values measured by the IC and ACCU was within 0.5 mm Al.^17^ Although the maximum error within the range of measurement conditions was −9%, which corresponded to an HVL of 0.5 mm Al. In addition, for tube voltages exceeding or equal to 80 kV and when the additional filter was thin, the change in the HVL obtained using the IC did not result in a smooth curve (Figure 4a). Therefore, at some measurement points, the value displayed by the ACCU was smaller than the lower limit of uncertainty for the IC (≥90 kV for Al 0, 2, 4, and Al 2 + Cu 0.1). In other words, it is necessary to recognize that a few errors may exist in the measurements obtained using the IC.

### 4.2 Dose rate and energy dependence of the SSD

For instruments that operate based on ionization, the influence on a larger error in the measured air kerma based on a correlation between the dose rate and the pulse height of the ionizing voltage has been reported.^27^ From this perspective, we examined the RDs in the dose rates. Because the dose rate under verified conditions were within a measurable range for ACCU, the present dose rate could be fairly evaluated. Consequently, no dose‐rate dependency was observed in the air‐kerma measurements but was observed in the tube voltage and HVL. This result suggested that the measurements obtained using the ACCU may indicate higher tube voltages and lower HVL readings with short‐term loads of increased tube current.

Although air kerma and tube voltage showed an energy dependence similar to that of the dose rate, HVL demonstrated a positive correlation. This result indicated that the RDs tended to approach zero as the radiation quality became harder. An energy uniformity of approximately 0.5 was used in the verification; however, the RQR5 standards mandated a 0.71, indicating that the x‐ray beam contained a considerably higher proportion of high‐energy components. Therefore, we believe that the difference between the calibration standard test conditions of the ACCU and the measurement conditions in this study affected the accuracy. Therefore, when a new SSD is used for elucidating the x‐ray irradiation conditions for patients, it is crucial to recognize the differences in the calibration and measurement beam conditions and thoroughly confirm the results of mutual comparisons with reference devices.

## AUTHOR CONTRIBUTIONS

All authors contributed to the study design and data interpretation. Experimental measurements were conducted by Hiroki Saito and Sho Maruyama. Sho Maruyama wrote the article and contributed to the entire study procedure as the principal investigator.

## CONFLICT OF INTEREST STATEMENT

The authors declare no conflicts of interest.

## COMPLIANCE WITH ETHICAL STANDARDS

This article does not contain any studies with human participants performed by any of the authors.

## Data Availability

The measured data that support the findings of this study are available from the corresponding author upon reasonable request.
